# Alzheimer's Therapeutic Strategy: Photoactive Platforms for Suppressing the Aggregation of Amyloid β Protein

**DOI:** 10.3389/fchem.2020.00509

**Published:** 2020-07-21

**Authors:** Chenglong Li, Jie Wang, Lei Liu

**Affiliations:** School of Material Science and Engineering, Institute for Advanced Materials, Jiangsu University, Zhenjiang, China

**Keywords:** photodynamic therapy, neurodegenerative disease, phostosensitive agent, amyloid protein, photocatalyst

## Abstract

Neurodegenerative diseases such as Alzheimer's disease (AD) have become a public health problem. Progressive cerebral accumulation of amyloid protein (Aβ) was widely considered as the cause of AD. One promising strategy for AD preclinical study is to degrade and clear the deposited amyloid aggregates with β-sheet-rich secondary structure in the brain. Based on the requirement, photo-active materials with the specific excitation and the standardization of the photosensitizer preparation and application in clinics, have attracted increased attention in the study and treatment of neurodegenerative disease as a novel method termed as photodynamic therapy (PDT). This review will focus on the new photosensitizing materials and discuss the trend of PDT techniques for the possible application in the treatment strategy of amyloid-related diseases.

## Introduction

Globally, especially in China, neurodegenerative diseases such as Alzheimer's disease (AD) have become a public health problem. Scientific studies confirmed that the abnormal aggregation of amyloid proteins was an important cause of pathology of amyloid-related diseases. Therefore, based on the hypothesis of β-amyloid, basic research and clinical medicine take amyloidosis as the treatment target to inhibit and reverse the self-assembly and aggregation of amyloid proteins. The environmental temperature (Zhang et al., 2018), pH value, and metal ions could modulate the aggregation of amyloid proteins. A series of amyloid inhibitors were also discovered, such as small compound molecules (Stachel et al., 2004; Reinke and Gestwicki, 2007), nanoparticles (Liao et al., 2012; Shaw et al., 2012), and biological macromolecules (Gobbi et al., 2010; Hadavi and Poot, 2016). In 2014, Qu xiaogang et al. found that polyoxometalate (POM) with wells Dawson structure effectively inhibited Aβ fibrillization, and these compounds can cross the blood-brain-barrier (BBB) (Gao et al., 2014). In 2019, Guo et al. discovered that the combination of calixarene and cyclodextrin can be used to inhibit the aggregation of amyloid protein (Xu et al., 2019). In recent years, Liu Lei's team has successively discovered a variety of inorganic nanomaterials, such as graphene oxide, carbon nanotubes, carbon quantum dots and graphene-like molybdenum disulfide nanosheet (MoS_2_), to exhibit certain inhibition effects on the aggregation of amyloid (Wang et al., 2015, 2016, 2018). At present, many inhibitors of amyloid aggregation have been reported, but most of them are far away from practical clinical treatment. There are still some issues that hinder the inhibitors' ability to be applied in treatment of the aggregation of amyloid-related disease *in vivo*: (1) the aggregation state of amyloid protein is favorable in thermodynamics, and most of the known inhibitors of amyloid protein can merely slow down or terminate the aggregation process of amyloid protein but not degrade the existing amyloid deposits without external energy input; (2) it is difficult to apply a temporospatial control over the traditional treatment duration and location *in vivo*.

Photodynamic therapy (PDT) has been put into medical use for a long period ever since the 1960s when Lipson and Baldes reported that porphyrin mixture containing neoplastic tissues could emit fluorescence under ultraviolet (Kessel, 2004). However, the porphyrin mixture named as hematoporphyrin derivative (HpD) was later found to have a strong phototoxicity for the tumor treatment. These early studies were quickly expanded into preclinical and clinical studies in the 1970s. Until now the first FDA-approved photosensitizer, Photofrin, quickly established a series of standards of clinical application around the world in the 1980s which laid a solid foundation of modern PDT. In clinical practice, the patient is injected intravenously with a photosensitizer. Then the tumor area is illuminated with an illumination intensity 25–50 J/cm. The side effect usually observed is a skin photosensitivity in patients which persists for a month or so. Of late, because of the advantages including minimal invasiveness and good temporospatial controllability, PDT has attracted the extensive interests of the researchers for the neurodegenerative diseases. PDT has been further applied to inhibit the aggregation of amyloid proteins. The photosensitizers (PS) with different excitation light play an important role in the PDT. In this review, we describe the applications of light-triggered agents for the anti-aggregation of Aβ proteins. With the development of PS and light sources, PDT applications could be categorized into three groups: ultraviolet (UV), visible light VIS) and near infrared light (NIR), which showed up almost in a chronological order.

As listed in Table 1, the light-responsive agents applied in PDT for AD with a series of light wavelengths as well as the illumination time, varied according to the optical characteristics of the photosensitizers.

**Table 1:** Photo-active agents and their applications in the PDT for AD.

**Classification**	**Photosensitizer**	**Publication time**	**Excitation light (wavelength)**	**Target**	**Incubation time**	**Biological model**	**References**
Ultraviolet	Fullerene–sugar hybrid	2010	UV (365 nm)	Aβ42 M, O	2 h		Ishida et al. (2010)
Light	Polyoxometalate	2013	UV (365 nm)	Aβ40 M, O	2 h	PC12	Li et al. (2013)
	Ag-TiO2	2013	315 nm	Aβ42 M, F	0.5–2 h	PC12	Ahmed et al. (2013)
	1,2,4-oxadiazole	2015	UV (260 nm)	Aβ40 M	5–24 h	LAN5	Mangione et al. (2015)
	Porphyrins-KLVFF	2014	UV (365 nm)	Aβ42 M, O	2 h	PC12	Hirabayashi et al. (2014)
Visible	Thioflavin T	2010	(442 nm)	Aβ40 F	24–40 h		Yagi et al. (2010)
Light	Tetra(4-sulfonatophenyl) porphyrin	2015	Blue (450 nm)	Aβ40 F	24 h	*Drosophila*	Lee et al. (2015a)
	Riboflavin	2014	White	Aβ42 M	1–6 h	PC12	Taniguchi et al. (2014)
	Rose bengal	2015	Green (525 nm)	Aβ42 M	12–24 h	PC12	Lee et al. (2015a)
	g-C3N4	2016	White	Aβ42 M	24 h	PC12	Chung et al. (2016)
	Polymeric micelles	2016	655 nm Laser	Aβ42 M F	24 h	PC 12	Zhang et al. (2016)
	Methylene Blue	2017	Red LED	Aβ42 F	15 min−24 h	*Drosophila*	Lee et al. (2017)
	CoPi@hematite	2017	white	Aβ42 M	3 h	PC 12	Kim et al. (2017)
	Ruthenium(II) {[Ru(bpy)3] 2+}	2017	white, red LED	Aβ42 F	24 h	PC 12	Son et al. (2018)
	Bismuth vanadate	2018	white LED	Aβ42 F	3 h	PC 12	Kim et al. (2018a,b)
		2019	630 nm	Aβ fibril	40 min	AD mice	Yue et al. (2019)
	Zn phthalocyanine	2019	665 nm LED	Aβ40 F	30 min	PC12	Zhan et al. (2019)
	Porphyrinic metal-organic frameworks	2019	450 nm	Aβ40 F	45 min	*C. elegans*	Yu et al. (2019)
NIR	RB/UCNP@ROS	2017	980 nm	Aβ42 F	12 h	PC12	Kuk et al. (2017)
light	βNaYF4:Yb/Er@SiO2@RB	2018	980 nm	Aβ42 F	48 h		Zhang et al. (2019)

*M, monomers; O, oligomers; F, pre-formed fibrils*.

The utilization of light-assisted therapy has attracted substantial interests for its merits of low side effects and noninvasiveness compared to traditional treatments of chemotherapy and surgery. For PDT, ultraviolet or visible light-excited photosensitizers could bring a limited penetration depth in the human tissues. In contrast, NIR light possesses a superior tissue penetration depth because most human tissues and fluids have the lowest adsorption of light within the wavelength of 700–1100 nm. Herein, the photo-responsive materials which can effectively absorb the NIR as an excitation light would be attractive for therapeutic applications.

## UV Excitation-Based Strategy

In 2010 Yagi et al. studied the irradiation of a laser on K3 amyloid fibrils. The light irradiation destroyed the existing fibrils and caused a partial destruction of growing fibrils. Further, a following explosive propagation after the irradiation took place, which was ascribed to the increment of the irradiation-induced active ends of proteins. The fragmentation of amyloid aggregates mainly depended on active oxygen radicals which reacted with the backbone of amyloid peptide (Yagi et al., 2010). After that they further combined the laser irradiation with the thioflavin T (ThT) to degrade the amyloid fibrils, which endowed the light irradiation specificity toward the amyloid protein deposits. The direct observation demonstrated that amyloid fibrils were fragmented by the laser irradiation. This result suggests the early successful application of laser-assisted destruction of amyloid fibrils (Ozawa et al., 2011). A similar study for anti-amyloid was completed by a fullerene-sugar hybrid composed of photoactive fullerene moiety and short peptide KLVFF, a core fragment of Aβ42. The hybrid inhibited Aβ peptide monomer and degraded the oligomers under a long-wavelength UV radiation condition, which is the first example of PDT application in the degradation of Aβ (Ishida et al., 2010). Several implications of UV-activated photosensitizer, such as Polyoxometalate (Li et al., 2013) and Ag-TiO_2_ (Ahmed et al., 2013), achieved similar anti-amyloid effects. All of those photosensitizers as inorganic agents could bind and degrade Aβ monomers and oligomers under UV irradiation conditions, providing typical examples of screening and designing inorganic PDT agents for amyloid proteins. Because the photo irradiation lowers the requirement of agent concentration, even the toxic photo-active agents could be safe for cells as confirmed by Polyoxometalate in cell tests. However, the limitation of UV (invasive energy) hindered its clinical applications. Visible light irradiation could substitute the UV light. The mechanism of PDT by UV and VIS is quite similar, and reactive oxygen species play an important role. In brief, photochemical reactions of photosensitizer with light irradiation generate various types of reactive oxygen, such as superoxide anions and hydroxyl radicals, etc. These radicals can react with the backbone of amyloid peptides and are responsible for the fragmentation and degradation.

## VIS Excitation-Based Photoactive Agents

Mostly after 2015, in order to increase the penetration depth and to mitigate the phototoxicity of UV, a variety of novel photosensitizers photo-activated by the visible light were synthesized and put into use for the inhibition and degradation of amyloid proteins. The photoactive agents were mainly composed of natural organic dyes (e.g., porphyrin and its derivatives) and metal oxide photocatalyst.

**Figure 1:**
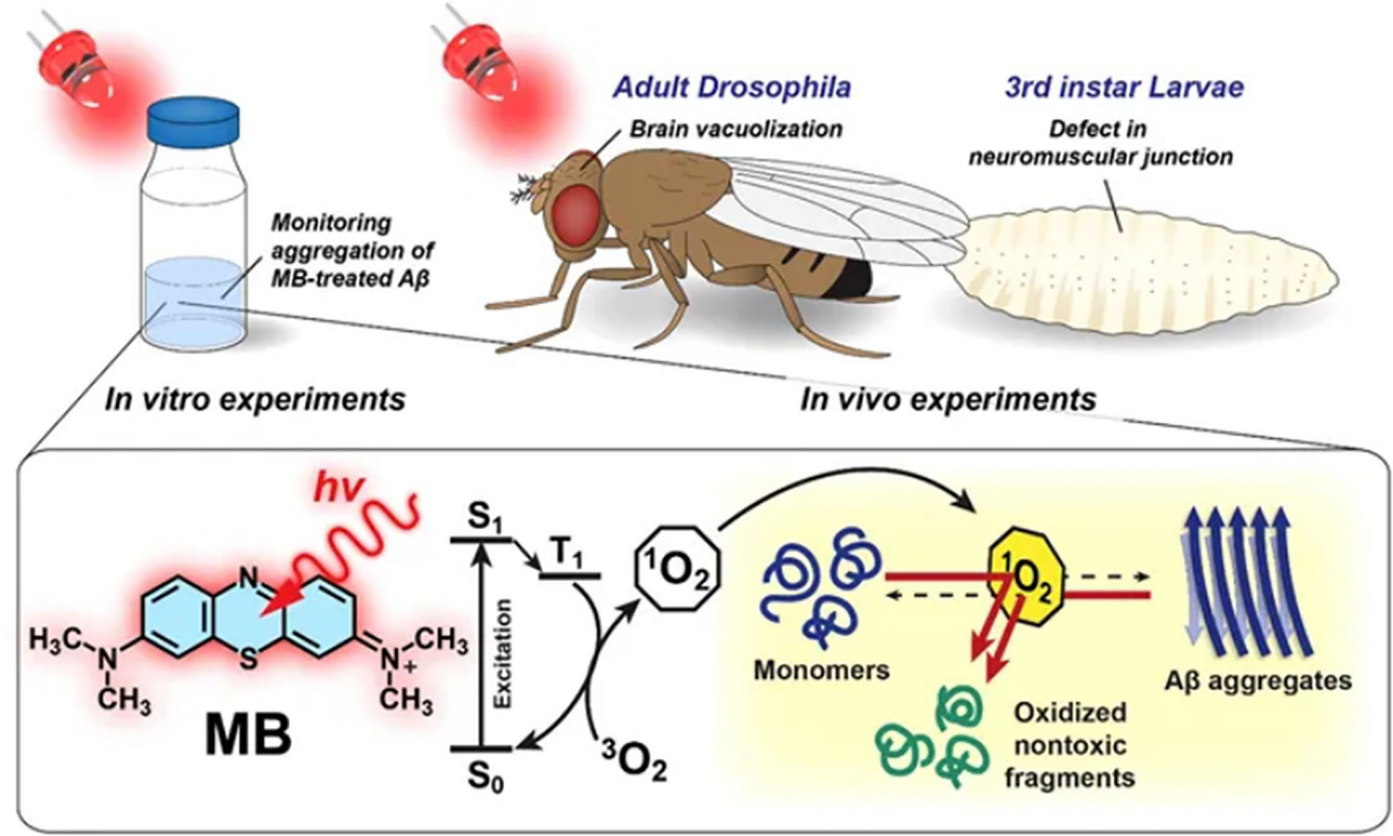
An illustration of dissociation of Aβ42 aggregates by photo-excited MB. Reproduced with permission (Lee et al., 2017). Copyright 2017, Nature Publishing Group.

In 2015, Lee et al. discovered rose bengal's strong inhibition effect on Aβ42 aggregation under a green LED. When binding with Aβ it could exhibit an enhancement and a remarkable red shift of the fluorescence emission. A mitigation effect for the cytotoxicity of Aβ42 for PC12 cells was confirmed *in vitro* (Lee et al., 2015b). At the same time, a porphyrin derivative, meso-tetra (4-sulfonatophenyl) porphyrin (TPPS) was reported by Byung et al. to inhibit Aβ aggregation under blue light illumination. Its mitigation of the cytotoxicity of Aβ was confirmed in the neuron cells and *Drosophila* AD model (Lee et al., 2015a). Later in 2017 they further utilized another dye methylene blue (MB), as shown in Figure 1, to disintegrate Aβ42 fibrils under the irradiation of red light with a longer light wavelength (630 nm) (Lee et al., 2017). The anti-Aβ effect of photoexcited MB fully rescued the AD phenotype *in vivo* using the *Drosophila* AD model. Particularly MB has a remarkable merit to cross brain-blood-barrier (BBB) facilitating its delivery to the confined brain area, which renders it a great potential to be used in treatment (Lee et al., 2017). The above-mentioned natural dyes and their derivatives are cheap and facile, but their biosafety and photo-degradation ability still have to be evaluated in mammalian animal models over a long period.

After 2017, a variety of metal oxide photocatalyst were designed and investigated for photodegrading the amyloid aggregates. Giyeong et al. reported visible light-driven dissociation of amyloid aggregates by ruthenium (II) complex {[Ru(bpy)_3_]^2+^}, which had been extensively applied in solar energy harvesting and conversion. It has a highly stable and long lifetime photoexcited state in liquid facilitating the efficient oxygen quenching and singlet oxygen production. In the experiment, ruthenium (II) complex successfully disassembled amyloid fibrils through oxidative damage caused by reactive oxygen species under white light-emitting diode (LED) light, which rescued the cytotoxicity of Aβ *in vitro* tests. Recent studies demonstrated Ru (II) polypyridyl complexes showed low cytotoxicity against normal fibroblast cells. The *in vivo* feasibility of these materials using AD animal models still needed to be examined in the future (Son et al., 2018). Yu et al. synthesized porphyrinic metal-organic frameworks (PMOFs) as excellent Cu (II) chelating agents to clear Aβ fibrils by the effective photochemical oxidation. Hf-MOFs have been demonstrated to be the most efficient PMOF to remove Aβ aggregates by photooxidation. The LPFFD-modified Hf-MOFs can alleviate Aβ toxicity and lengthen the lifespan of *C. elegans* CL2006, which is a model animal of AD. This work cast insights into the specially-designed MOFs as neurotoxic-metal chelating and PDT agent (Yu et al., 2019).

Except for the above trials, a few photo-responsive nanomaterials and polymers also showed the photo-degradation effect on amyloid protein deposits. Graphitic carbon nitride (g-C_3_N_4_) was usually applied in solar water-splitting and hydrogen generation for its chemical and thermal stabilities with a narrow bandgap of 2.6–2.8 eV. In 2016 based on its photocatalysis ability, g-C_3_N_4_ was utilized to inhibit Aβ aggregation under a white LED. The effect of Ag-doping g-C_3_N_4_ on the anti-Aβ aggregation was also investigated, which proposed a regime for designing new photosensitizer based on the inorganic photocatalyst (Chung et al., 2016). Zhang et al. combined a drug delivery platform of diblock polymer and a photosensitizer, chlorin e6(Ce6) to develop a novel micell for degrading the toxic Aβ oligomers and protofibrils under a 655 nm laser. The PEG-b-PDPA copolymer micelle conjugated with Ce6 was also loaded with an Aβ inhibitor Tanshinone I (TAS). TAS can prevent Aβ fibrillization and upon laser irradiation Ce6-conjugated micelles can degrade Aβ aggregates by generating singlet oxygen. Given the potentials of a 655 nm laser and the micelle for penetrating the BBB, the nanocomposite provided an optional insight for AD therapy (Zhang et al., 2016).

The nature of photoactive inorganic agents is usually hard and hydrophobic, which is not compatible for the soft and hydrophilic human tissues. For example, g-C_3_N_4_ is a hydrophobic nanomaterial and is usually not stable in the PBS solution. For realistic application in the brain, it is desirable to perform surface modification on these inorganic agents, such as the addition of targeting peptides and PEG polymers. Furthermore, at present FDA-approved PDT with 630 nm laser has the penetration depth of several millimeters for most human tissues. For a clinical application, a miniaturized optical fiber integrated with a microscale injectable illumination module is required, due to the limit of penetration depth of photosensitizers with the photoexcitation of UV-Vis light.

## NIR Excitation-Based Photoactive Agents

In the development of photoactive treatment,UV and visible light in the PDT were suffered from an irradiation damage to normal tissues and a limited penetration depth (Peng et al., 2011). For most human tissues, the long-wavelength NIR has at least 2-fold penetration depth compared with UV-Vis. To overcome these obstacles, rare earth-based upconversion nanoparticles (UCNPs) have been proposed as an energy transducer to activate PS. For the past few years, UCNPs attracted considerable attention for its ability to generate short wavelength fluorescence (visible or UV light) by utilizing NIR light irradiation via two photons or multiphoton mechanism (Nyk et al., 2008). In order to generate ROS, the UCNPs and photosensitizers have been combined for photodynamic therapy (Maji et al., 2014; Mei et al., 2015; Ai et al., 2016). Ni et al. fabricated a NIR-excited nanoprobe (ANG/PEG-UCNPs) which can cross the BBB functioning as a MR/fluorescence bimodal imaging agent with a superior performance under a NIR photo-activation. Moreover, the toxicity, especially to brains, was assessed to be negligible *in vivo* (Ni et al., 2014). Lu et al. utilized a silicon-coating on UCNPs to enhance the energy transfer from NIR light by providing an interior cavity for high payload of photosensitizers rose bengal at an absorbance overlapping with the visible emission of Er^3+^ (Lu et al., 2015). In 2019, we synthesized β-NaYF4:Yb/Er@SiO_2_@RB by conjugating UCNPs with rose Bengal to degrade preformed Aβ aggregates under a 980 nm NIR light. The results revealed that UCNPs could effectively photo-degrade Aβ42 fibrils, which demonstrated UCNP's potential application in the treatment of amyloid diseases (Yu et al., 2019). The development of NIR photosensitizers for degenerative disease is just in its infancy. Several issues have to be addressed urgently for future therapeutic applications, e.g., the metabolic pathways, cytotoxicity and the side-effect of accumulated ROS have not been completely investigated *in vivo* studies especially with mammal animal models, which is essential to assess clinical applicability.

## Perspective

Nowadays photosensitizers and approved clinical indications of PDT exist in many countries though the total number is still limited especially for brain disorders. To date, there is no standard method for evaluating the efficiency of the photosensitizers. Most researches rely on *in vitro* techniques such as AFM, TEM, CD, ThT fluorescence, electrophoresis, etc. The biosafety validation counts on the cell experiment and *in vivo* experiments with *Drosophila* and *C. elegans* AD models. There is still a big gap for the pre-clinical treatment, e.g., the penetration range for NIR-excited photosensitizers in human brain, the metabolic pathways of the PS and the disintegrated amyloids, and most PDT applications have not been tested on the mammal model for their feasibility, such as ability to cross the BBB and the long-term toxicity effects.

The translation of PDT to pathologies that are deeply situated in the brain is primarily restricted by the limited penetration depth of light. At present, most clinical applications of PDT have been limited to superficial layers of tissues, such as the skin and retina. The delivering of photoexcitation to deep target tissues (e.g., brain tumors) has been restricted by a significant attenuation of light irradiation during the penetration process (Mallidi et al., 2016). In past years, the light sources of PDT applications have gradually evolved from non-coherent to laser systems that allow excitation light at a given wavelength to be delivered into a confined region which would benefit its clinical use. Recent progresses in optogenetics, which could control a specific neural activity in the brain circuit with a cast of light, facilitate the temporospatial controllability of PDT for neurodegenerative diseases. Furthermore, the light guides such as fiber optics, which could transfer the light flux to the deep tissue areas, could be utilized to illuminate the confined brain area and activate (or reversely deactivate) a specific neural circuit (Dugué et al., 2012). It has been observed and expected that the arising photodynamic agents with excellent biocompatibility and the rapid development of wireless and implantable micro LED may bring breakthrough to the existing barriers in photoactive treatment of amyloid diseases (Rossi et al., 2015).

## Author Contributions

CL and JW contributed equally to the literature search and analysis, data integration, and writing the manuscript. LL instructed and wrote the manuscript. All authors contributed to the article and approved the submitted version.

## Conflict of Interest

The authors declare that the research was conducted in the absence of any commercial or financial relationships that could be construed as a potential conflict of interest.

## Abbreviations

PDT: Photodynamic therapy
PS: Photosensitizer
BBB: Blood-brain-barrier
UCNP: Upconversion nanoparticle.

## References

[B1] Ahmed M. H. Keyes T. E. Byrne J. A. (2013). The photocatalytic inactivation effect of Ag-TiO_2_ on β-amyloid peptide (1-42). J. Photochem. Photobiol A. 254, 1–11. 10.1016/j.jphotochem.2012.12.019

[B2] Ai X. Ho C. J. H. Aw J. Attia A. B. E. Mu J. Wang Y.. (2016). *In vivo* covalent cross-linking of photon-converted rare-earth nanostructures for tumour localization and theranostics. Nat. Commun. 7, 1–9. 10.1038/ncomms10432 26786559 PMC4736106

[B3] Chung Y. J. Lee B. I. Ko J. W. Park C. B. (2016). Photoactive g-C3N4 nanosheets for light-induced suppression of Alzheimer's β-amyloid aggregation and toxicity. Adv. Healthcare Mater. 5, 1560–1565. 10.1002/adhm.201500964 27111552

[B4] Dugué G. P. Akemann W. Knöpfel T. (2012). A comprehensive concept of optogenetics. Prog. Brain Res. 196, 1–28. 10.1016/B978-0-444-59426-6.00001-X 22341318

[B5] Gao N. Sun H. Dong K. Ren J. Duan T. Xu C.. (2014). Transition-metal-substituted polyoxometalate derivatives as functional anti-amyloid agents for Alzheimer's disease. Nat. Commun. 5:4422. 10.1038/ncomms4422 24595206

[B6] Gobbi M. Re F. Canovi M. Beeg M. Gregori M. Sesana S.. (2010). Lipid-based nanoparticles with high binding affinity for amyloid-β1-42 peptide. Biomaterials 31, 6519–6529. 10.1016/j.biomaterials.2010.04.044 20553982

[B7] Hadavi D. Poot A. A. (2016). Biomaterials for the treatment of Alzheimer's disease. Front. Bioeng. Biotech. 4:49. 10.3389/fbioe.2016.00049 27379232 PMC4909781

[B8] Hirabayashi A. Shindo Y. Oka K. Takahashi D. Toshima K. (2014). Photodegradation of amyloid β and reduction of its cytotoxicity to PC12 cells using porphyrin derivatives. Chem. Commun. 50, 9543–9546. 10.1039/C4CC03791J 25012260

[B9] Ishida Y. Tanimoto S. Takahashi D. Toshima K. (2010). Photo-degradation of amyloid β by a designed fullerene-sugar hybrid. Med. Chem. Commun. 1, 212–215. 10.1039/c0md00075b

[B10] Kessel D. (2004). Photodynamic therapy: from the beginning. Photodiagn. Photodyn. 1, 3–7. 10.1016/S1572-1000(04)00003-1 25048058

[B11] Kim K. Lee B. I. Chung Y. J. Choi W. S. Park C. B. (2017). Hematite-based photoelectrode materials for photoelectrocatalytic inhibition of Alzheimer's β-amyloid self-assembly. Adv. Healthc Mater. 6:1601133. 10.1002/adhm.201601133 28194907

[B12] Kim K. Lee S. H. Choi D. S. Park C. B. (2018a). Alzheimer's disease: photoactive bismuth vanadate structure for light-triggered dissociation of Alzheimer's β-amyloid aggregates. Adv. Funct. Mater. 28:1870298 10.1002/adfm.201870298

[B13] Kim K. Lee S. H. Choi D. S. Park C. B. (2018b). Photoactive bismuth vanadate structure for light-triggered dissociation of Alzheimer's β-amyloid aggregates. Adv. Funct. Mater. 28:1802813 10.1002/adfm.201802813

[B14] Kuk S. Lee B. I. Lee J. S. Park C. B. (2017). Upconversion nanoparticles: rattle-structured upconversion nanoparticles for near-IR-induced suppression of Alzheimer's β-amyloid aggregation. Small 13:1603139. 10.1002/smll.201603139 28092125

[B15] Lee B. I. Lee S. Suh Y. S. Lee J. S. Kim A. K. Kwon O. Y. (2015a). Photoexcited porphyrins as a strong suppressor of β-amyloid aggregation and synaptic toxicity. Angew. Chem. Int. Edit. 54, 11472–11476. 10.1002/anie.201504310 26178411

[B16] Lee B. I. Suh Y. S. Chung Y. J. Yu K. Park C. B. (2017). Shedding light on Alzheimer's β-amyloidosis: photosensitized methylene blue inhibits self-assembly of β-amyloid peptides and disintegrates their aggregates. Sci. Rep. 7:7523. 10.1038/s41598-017-07581-2 28790398 PMC5548810

[B17] Lee J. S. Lee B. I. Park C. B. (2015b). Photo-induced inhibition of Alzheimer's β-amyloid aggregation *in vitro* by rose bengal. Biomaterials 38, 43–49. 10.1016/j.biomaterials.2014.10.058 25457982

[B18] Li M. Xu C. Ren J. Wang E. Qu X. (2013). Photodegradation of β-sheet amyloid fibrils associated with Alzheimer's disease by using polyoxometalates as photocatalysts. Chem. Commun. 49, 11394–11396. 10.1039/c3cc46772d 24165705

[B19] Liao Y. H. Chang Y. J. Yoshiike Y. Chang Y. C. Chen Y. R. (2012). Negatively charged gold nanoparticles inhibit Alzheimer's amyloid-β fibrillization, induce fibril dissociation, and mitigate neurotoxicity. Small 8, 3631–3639. 10.1002/smll.201201068 22915547

[B20] Lu S. Tu D. Hu P. Xu J. Li R. Wang M.. (2015). Multifunctional nano-bioprobes based on rattle-structured upconverting luminescent nanoparticles. Angew. Chem. Int. Edit. 54, 7915–7919. 10.1002/anie.201501468 26013002

[B21] Maji S. K. Sreejith S. Joseph J. Lin M. He T. Tong Y.. (2014). Upconversion nanoparticles as a contrast agent for photoacoustic imaging in live mice. Adv. Mater. 26, 5633–5638. 10.1002/adma.201400831 24913756

[B22] Mallidi S. Anbil S. Bulin A. L. Obaid G. Ichikawa M. Hasan T. (2016). Beyond the barriers of light penetration: strategies, perspectives and possibilities for photodynamic therapy. Theranostics 6, 2458-2487. 10.7150/thno.16183 27877247 PMC5118607

[B23] Mangione M. R. Piccionello A. P. Marino C. Ortore M. G. Picone P. Vilasi S. (2015). Photo-inhibition of Aβ fibrillation mediated by a newly designed fluorinated oxadiazole. RSC Adv. 5, 16540–16548. 10.1039/C4RA13556C

[B24] Mei Q. Deng W. Yisibashaer W. Jing H. Du G. Wu M.. (2015). Zinc-dithizone complex engineered upconverting nanosensors for the detection of hypochlorite in living cells. Small 11, 4568–4575. 10.1002/smll.201501130 26150405

[B25] Ni D. Zhang J. Bu W. Xing H. Han F. Xiao Q.. (2014). Dual-targeting upconversion nanoprobes across the blood-brain barrier for magnetic resonance/fluorescence imaging of intracranial glioblastoma. ACS Nano 8, 1231–1242. 10.1021/nn406197c 24397730

[B26] Nyk M. Kumar R. Ohulchanskyy T. Y. Bergey E. J. Prasad P. N. (2008). High contrast *in vitro* and *in vivo* photoluminescence bioimaging using near infrared to near infrared up-conversion in Tm3^+^ and Yb3^+^ doped fluoride nanophosphors. Nano Lett. 8, 3834–3838. 10.1021/nl802223f 18928324 PMC3523349

[B27] Ozawa D. Kaji Y. Yagi H. Sakurai K. Kawakami T. Naiki H.. (2011). Destruction of amyloid fibrils of keratoepithelin peptides by laser irradiation coupled with amyloid-specific thioflavin T. J. Biol. Chem. 286, 10856–10863. 10.1074/jbc.M111.222901 21300800 PMC3060536

[B28] Peng C. Li Y. Liang H. Cheng J. Li Q. Sun X.. (2011). Detection and photodynamic therapy of inflamed atherosclerotic plaques in the carotid artery of rabbits. J. Photochem. Photobiol., B 102, 26–31. 10.1016/j.jphotobiol.2010.09.001 20875747

[B29] Reinke A. A. Gestwicki J. E. (2007). Structure-activity relationships of amyloid beta-aggregation inhibitors based on curcumin: influence of linker length and flexibility. Chem. Biol. Drug. Des. 70, 206–215. 10.1111/j.1747-0285.2007.00557.x 17718715

[B30] Rossi M. A. Go V. Murphy T. Fu Q. Morizio J. Yin H. H. (2015). A wirelessly controlled implantable LED system for deep brain optogenetic stimulation. Front. Integr. Neurosc. 9:8. 10.3389/fnint.2015.00008 25713516 PMC4322607

[B31] Shaw C. P. Middleton D. A. Volk M. Lévy R. (2012). Amyloid-derived peptide forms self-assembled monolayers on gold nanoparticle with a curvature-dependent β-sheet structure. ACS Nano 6, 1416–1426. 10.1021/nn204214x 22242947

[B32] Son G. Lee B. I. Chung Y. J. Park C. B. (2018). Light-triggered dissociation of self-assembled β-amyloid aggregates into small, nontoxic fragments by ruthenium (II) complex. Acta Biomater. 67, 147–155. 10.1016/j.actbio.2017.11.048 29221856

[B33] Stachel S. J. Coburn C. A. Steele T. G. Jones K. G. Loutzenhiser E. F. Gregro A. R.. (2004). Structure-based design of potent and selective cell-permeable inhibitors of human β-secretase (BACE-1). J. Med. Chem. 47, 6447–6450. 10.1021/jm049379g 15588077

[B34] Taniguchi A. Sasaki D. Shiohara A. Iwatsubo T. Tomita T. Sohma Y.. (2014). Attenuation of the aggregation and neurotoxicity of amyloid-β peptides by catalytic photooxygenation. Angew. Chem. Int. Edit. 53, 1382–1385. 10.1002/anie.201308001 24339209

[B35] Wang J. Cao Y. Li Q. Liu L. Dong M. (2015). Size effect of graphene oxide on modulating amyloid peptide assembly. Chem. Eur. J. 21, 9632–9637. 10.1002/chem.201500577 26031933

[B36] Wang J. Liu L. Ge D. Zhang H. Feng Y. Zhang Y.. (2018). Differential modulating effect of MoS_2_ on the assemblies of amyloid peptides. Chem. Eur. J. 24, 3397–3402. 10.1002/chem.201704593 29210123

[B37] Wang J. Zhu Z. Bortolini C. Hoffmann S. V. Amari A. Zhang H. X.. (2016). Dimensionality of carbon nanomaterial impacting on the modulation of amyloid peptide assembly. Nanotechnology 27:304001. 10.1088/0957-4484/27/30/304001 27302044

[B38] Xu Z. Jia S. Wang W. Yuan Z. Ravoo B. J. Guo D.-S. (2019). Heteromultivalent peptide recognition by co-assembly of cyclodextrin and calixarene amphiphiles enables inhibition of amyloid fibrillation. Nat. Chem. 11, 86–93. 10.1038/s41557-018-0164-y 30455432

[B39] Yagi H. Ozawa D. Sakurai K. Kawakami T. Kuyama H. Nishimura O.. (2010). Laser-induced propagation and destruction of amyloid fibrils. J. Biol. Chem. 285, 19660–19667. 10.1074/jbc.M109.076505 20406822 PMC2885244

[B40] Yu D. Guan Y. Bai F. Du Z. Gao N. Ren J.. (2019). Metal-organic frameworks harness Cu chelating and photooxidation against amyloid β aggregation *in vivo*. Chem. Eur. J. 25, 3489–3495. 10.1002/chem.201805835 30601592

[B41] Yue X. Mei Y. Zhang Y. Tong Z. Cui D. Yang J.. (2019). New insight into Alzheimer's disease: light reverses Aβ-obstructed interstitial fluid flow and ameliorates memory decline in APP/PS1 mice. Alzheimers Dement. 5, 671–684. 10.1016/j.trci.2019.09.007 31720368 PMC6838540

[B42] Zhan Q. Shi X. Wang T. Hu J. Zhou J. Zhou L.. (2019). Design and synthesis of thymine modified phthalocyanine for Aβ protofibrils photodegradation and Aβ peptide aggregation inhibition. Talanta 191, 27–38. 10.1016/j.talanta.2018.08.037 30262061

[B43] Zhang H. X. Liu L. Wang J. Bortolini C. Dong M. (2018). Thermal effect on the degradation of hIAPP20-29 fibrils. J. Colloid. Interf. Sci. 513, 126–132. 10.1016/j.jcis.2017.10.107 29145016

[B44] Zhang J. Liu J. Zhu Y. Xu Z. Xu J. Wang T.. (2016). Photodynamic micelles for amyloid β degradation and aggregation inhibition. Chem. Commun. 52, 12044–12047. 10.1039/C6CC06175C 27711295

[B45] Zhang Z. Wang J. Song Y. Wang Z. Dong M. Liu L. (2019). Disassembly of Alzheimer's amyloid fibrils by functional upconversion nanoparticles under near-infrared light irradiation. Colloid Surface B 181, 341–348. 10.1016/j.colsurfb.2019.05.053 31158696

